# Cellular and Molecular Mechanisms and Innovative Neurostimulation Treatments in the Management of Traumatic Brain Injury

**DOI:** 10.26502/jbb.2642-91280169

**Published:** 2024-11-14

**Authors:** Arnav Aggarwal, Yssel Mendoza-Mari, Devendra K Agrawal

**Affiliations:** 1 Loveless Academic Magnet Program, Montgomery, AL, USA; 2 Department of Translational Research, College of Osteopathic Medicine of the Pacific, Western University of Health Sciences, Pomona CA 91766, USA

**Keywords:** Deep brain stimulation, Pulsed electromagnetic field stimulation, Traumatic brain injury, Transcranial direct current stimulation, Transcranial magnetic stimulation

## Abstract

Traumatic brain injury (TBI) is one of the growing public health problems and a leading cause of disabilities and mortality worldwide. After the mechanical impact to the head, patients of all ages suffer from cognitive and neurological deficits, as well as psychological disorders to different extents. In the last years, the use of electrical impulses and magnetic currents to achieve therapeutic effects have shown promising results and became potential treatments for TBI. Potential mechanisms of action described so far include long term potentiation and depression of neuronal synapses, stimulation of neurotransmitters and growth factors release, and reduction of neuroinflammation, apoptosis and excitotoxicity, among others. Although promising results have been obtained in pre-clinical experiments and limited clinical studies, the high rate of variability in technical parameters and the limited number of patients enrolled have made difficult to clearly define the optimal conditions to obtain reliable therapeutic effects with these stimulation techniques in TBI patients. The present review aims to describe the molecular processes taking place in the brain after the injury, as well as to describe some of the neurostimulation treatments currently under development for TBI management.

## Introduction

Traumatic brain injury (TBI) is one of the leading causes of disabilities worldwide. Patients suffering from this injury develop serious complications leading to lower their quality of life. TBI can be classified based on: (i) the mechanism: open (broken fractured or penetrated skull) or closed (blunt) ; (ii) the severity: mild (brief loss of consciousness for a few seconds or minutes), moderate (loss of consciousness for hours), or severe (loss of consciousness or coma for more than a day) ; and (iii) other features (location of injury, etc.) [1]. According to the severity of the trauma, TBI patients develop a wild spectrum of sequelae that can last from days, several weeks or even months [2]. Cognitive impairments include planning, attention, memory and working memory, problem solving and decision-making, and production of language. Other symptoms include balance problems, headache, dizziness, fatigue, sleep disturbance, and emotional dysregulation like irritability, stress intolerance, light and sound sensitivity, anxiety, and depressed mood [3]. Rehabilitation, cognitive correction, exercise, and cognitive-enhancing drugs, as well as various brain stimulation techniques are currently used for treating TBI [4]. These approaches can be categorized into pharmacological [5] and nonpharmacological treatments [6]. Among the non- pharmacological methodologies, neuromodulatory techniques aim to alter pathological activities of the nervous system, through the use of electrical impulses and magnetic currents, to achieve therapeutic effects and have shown promising results [4]. Brain stimulation can alter the firing of neurons, boost synaptic strength, alter neurotransmitters and excitotoxicity, and modify the connections in their neural networks [7]. They can be also used to decrease the inflammation, increase the cerebral blood flow, modulate the activity of neurotrophic factors or neural excitability, and enhance the cortical function by facilitating long term potentiation (LTP) or reducing long term depression (LTD) responses to slow or reverse neurological decline [8]. Although a broad variation in clinical results has been obtained using these neuromodulatory techniques, the promising results observed in preclinical studies with electrical stimulation provide a good rationale for continue exploring this field to alleviate trauma-induced disabilities. In the present article we aim to give an overview on different molecular events arising after the mechanical impact to the head, which are responsible for the appearance of the vast myriads of symptoms observed in TBI patients, and secondly to describe some of the neuromodulatory interventions, their mechanisms of action and the biological processes they stimulate.

## Pathological Changes after Traumatic Brain Injury

During the primary phase of TBI, the mechanical impact on the head leads to scalp laceration and contusion, skull fracture, blood vessels damage, extra and intraparenchymal hemorrhages, and traumatic focal and diffuse axonal injuries. Focal brain damage is characterized by necrotic areas of neuronal and glial cells concentrated in the area of impact. Secondary contusion may appear in opposite areas to the primary collision due to brain rebounds and strikes inside the skull [9]. Although both types of damages co-exist in patients who suffered from moderate to severe TBI, diffuse axonal injury (DAI) is present in approximately 70% of TBI cases [10]. DAI is mediated by non-contact forces of rapid deceleration and acceleration, which cause shearing and stretching damage in cerebral brain tissues. The strong tensile forces impair neuronal axons, oligodendrocytes and blood vasculature, leading to brain edema and ischemic brain damage [11]. From the structural localization point of view, DAI usually occurs in the deep parasagittal white matter, internal capsule, corpus callosum, fornix and upper brain stem, while cortical contusions arise in the frontal and temporary lobes. TBI can also affect distal structures like the hypothalamus and the pituitary [12]. Axonal damage involves degradation of cytoskeleton microtubules and neurofilaments, and impaired transport and its persistence and extension define the severity of TBI [13]. The pathological changes that take place during the secondary phase of trauma include blood-brain barrier (BBB) disturbance, glutamate excitotoxicity, neuroinflammation, oxidative and nitrosative stress, mitochondrial dysfunction, cerebral edema, demyelination, decreased neurogenesis, among others [12]. These molecular events are directly involved in tissue damage, atrophy and cell death that are responsible for the development of cognitive and motor impairments, psychiatric disorders and other morbidities [14].

## Axonal degeneration

Immediately after mechanical trauma, the disorganization of neuronal microtubules and neurofilaments occurs comprising the axonal cytoskeletal network [13]. Acute axonal damage is characterized by myelin sheath degradation, axonal transport damage, and buildup of axonal transport proteins and, when persisting in time, progresses into a delayed secondary axotomy state [15]. The dissociation of axonal connections and the accumulation of axonal proteins promotes the formation of retraction bulbs, which comprises protein markers such as β-amyloid precursor protein and neurofilament, and results in prolonged swelling of injured axons and apoptotic cell death of neurons and oligodendrocytes [16].

## Excitotoxicity

BBB disruption induces the release of excess excitatory neurotransmitters such as glutamate and aspartate from pre-synaptic terminals [17]. Excessive glutamate causes overexpression of ionotropic (N-methyl-d-aspartate (NMDA) and a-amino-3-hydroxy-5-methyl-4- isoxazole propionate (AMPA)) and metabotropic glutamate receptors and alters ion homeostasis by permitting extracellular Ca^2+^ and Na^+^ ions to enter the cell, causing membrane depolarization in neurons [18]. High levels of intracellular Ca^2+^ trigger downstream signaling molecules such as Ca^2+^/calmodulin-dependent protein kinase II, mitogen-activated protein kinases (MAPK), protein phosphatases, and protein kinase C. Elevated cytosolic Ca^2+^ also activates apoptotic proteins such as calpain, calcineurin, and caspases [19] and increases the production of reactive oxygen species (ROS) and nitric oxide (NO) [20]. All these factors contribute to cell death.

## Mitochondrial dysfunction

Accumulation of Ca^2+^ and ROS inside the cytoplasm after TBI impairs mitochondrial function. Depolarization of mitochondrial membranes and inhibition of ATP synthesis lead to the failure of electron transport chain and oxidative phosphorylation processes [21]. Increased activity in membrane pumps, aiming to restore ionic balance, raises glucose consumption, depletes energy stores, causes Ca^2+^ influx into mitochondria, and impairs oxidative metabolism and consequently anaerobic glycolysis with lactate production, which might cause acidosis and edema [22]. Moreover, after TBI, mitochondrial permeability transition pore is also activated, which leads to an increase in inner membrane permeability. As consequence, mitochondrial proteins such as cytochrome c and apoptosis-inducible factor are released to the cytosol, contributing to apoptotic cell death [23].

## Apoptosis

The second phase of damage after TBI is typically characterized by the apoptotic cell death of neurons and oligodendrocytes. Several molecular pathways such as extracellular signal-regulated kinase, p38 MAPK, janus kinase/signal transducer and activator of transcription activate caspases and calpain. Caspase-dependent cell death could be triggered by the activation of extrinsic death receptor pathway or intrinsic mitochondrial pathway [24]. The extrinsic pathway is mediated by tumor necrosis factor alpha (TNF-α) and Fas, whereas intrinsic pathway relies on the release of cytochrome c after mitochondrial depolarization. In both cases, caspase 8 and caspase 9 activation lead to proteolytical cleavage and activation of effector caspase 3 [24]. On the other hand, caspase-independent apoptosis is mediated by calpain activation after the proteolysis of cytoskeletal proteins and the release of mitochondrial proteins such as apoptosis inducible factor. These mitochondrial proteins translocate into the nucleus and activate downstream signaling molecules, resulting in DNA damage and chromatin condensation in neuronal and glial cells [25].

## Neuroinflammation

Infiltration of circulating neutrophils, monocytes and lymphocytes into the injured brain parenchyma occurs because of BBB disruption and directly influences neuronal survival and death [26]. mRNA and protein levels of pro-inflammatory cytokines as interleukin (IL)-1β, IL-6, TNF-α, among others increase 4 to 6 h after trauma [27], as well as prostaglandins, damage associated molecular patterns, complement factors and free radicals [28]. Damaged cells release intracellular components into circulation and the extracellular space, such as heat shock proteins 60 and 70, nucleic acids, and high mobility group box protein B1 (HMGB1) that activate pattern recognition receptors for downstream cell signaling [29]. Other chemokines like IL-8, macrophage inflammatory protein 1 alpha, and monocyte chemoattractant protein 1, and endothelial and leukocyte cell adhesion molecules such as intercellular adhesion molecule 1 and vascular cell adhesion molecule 1 are also upregulated and contribute to further recruit leukocytes to the injury site [30]. Activation of microglia, astrogliosis, accumulation of macrophages and progressive phagocytosis due to prolonged neuroinflammation characterize the injured brains of patients and experimental animals [31]. The activated microglia help in clearing cell debris and promote tissue remodeling, but also release various neurotoxic substances, such as ROS, reactive nitrogen species, and excitatory neurotransmitters, such as glutamate, that may exacerbate neuronal death [32]. It is considered that neuroinflammation could be beneficial for TBI resolution, if can be regulated, or detrimental when becomes excessive and chronic, giving rise to the development of numerous neuropathologies [33].

## Oxidative stress

Oxidative stress is directly associated with mitochondrial dysfunction. Besides, excessive ROS and free radicals are generated from enzymatic processes, activated neutrophils, and excitotoxic pathways [34]. Intracellular accumulation of Ca^2+^ promotes the activity of NO synthases and the subsequent increased production of NO, which in turn reacts with free radical superoxide, forming peroxynitrite [35]. Unpaired electrons in ROS molecules facilitate their binding to nucleic acids, proteins and lipids, causing extended cellular damage. In particular, the association of ROS to polyunsaturated fatty acids in membrane phospholipids gives rise to lipoperoxyl radicals, impairing cell membrane integrity and functioning [36]. Oxidative stress also hinders synaptic plasticity in injured cortex and hippocampus, with the related loss of synaptic proteins [36]. High levels of ROS after TBI overloads the efficiency of the endogenous antioxidant systems such as glutathione reductase, glutathione-S-transferase, glutathione peroxidase, catalase, superoxide dismutase, and uric acid, tipping the balance towards a pro-oxidative state [37]. In general, exacerbated presence of ROS and lipid peroxidation negatively affects cerebral blood flow and brain plasticity, causes immunosuppression, DNA break, oxidation of proteins, and inhibition of mitochondrial respiration; all of them are contributing factors for apoptotic or necrotic cell death [38].

## TBI and Neuromodulation

The lack of efficient treatments to overcome the impact of TBI and minimize the appearance of further neurological complications has led to continuous search for novel therapeutic strategies. Among them, non-pharmacological interventions have been extensively explored in different pre-clinical scenarios (See reviewed information in [6]). One of the targeted mechanisms currently under study is neuromodulation. This term describes the alteration of neuronal activity through the delivery of a chemical or electrical stimulus to specific neural targets in the body, to promote neuronal plasticity and recovery from neurological disabilities [39]. Depending on the pathological condition or the neuronal area involved, hypo- or hyper-excitation of neurons constitutes target for neuromodulatory interventions. The ability of the human brain to find alternative signaling mechanisms, despite its low capacity to self-repair, can be fueled by electrical and magnetic stimulation, and this neuromodulation could be beneficial to improve neuroplasticity and connectivity. The excitability of neuronal cells facilitates modulation of their firing activity using external stimulation to enhance or suppress endogenous activity, contributing to the modification of structural arrangements and strength of synaptic connections, and ultimately to increase functional capacity [40]. One of the advantages of neurostimulation is that it is also effective in areas different from the injured one due to the interconnection among the brain regions [40].

The use of electrical impulses and magnetic currents to stimulate specific areas in the nervous system aims to reconfigure neuronal circuits that have been damaged due to external injuries or neurodegenerative disorders. In the case of TBI, the main goal of these non- pharmacological approaches is to modulate neural activity to promote functional reorganization. The deficits experienced by affected individuals depend on the severity of damage and the areas of the brain affected, and they may continue for years after the initial traumatic insult, leading to significant and long-lasting functional impairment [41]. Symptoms of single mild TBI comprise headache, dizziness, disorientation, and confusion [42]. But when it is repeated, the damage can result in cognitive impairment, short-term memory loss, increased impulsivity and aggression, and susceptibility to chronic traumatic encephalopathy [43]. Cognitive symptoms of mTBI might also include difficulty focusing attention and reduced visual processing speed. During moderate to severe TBI the patients also develop long-term motor and cognitive disabilities including problems with memory, attention, and the regulation of emotions [44] and, in some cases, they also suffer from major depression and/or sleep disturbances such as insomnia, narcolepsy, and excessive daytime sleepiness [45]. From an anatomical point of view, some studies have revealed altered structural integrity of white matter [46] and corpus callosum of patients with mild and moderate TBI [47] due to brain injury-induced demyelination [48]. Other regions involved are hippocampus and prefrontal cortex [49]. Based on the wide variety of symptoms, it can be deduced that numerous neural circuits are compromised in the damage. Therefore, the use of therapeutic approaches that reach local and distant damaged areas in the brain could be very useful to minimize the impact of TBI and increase the patient's quality of life.

**Figure 1:**
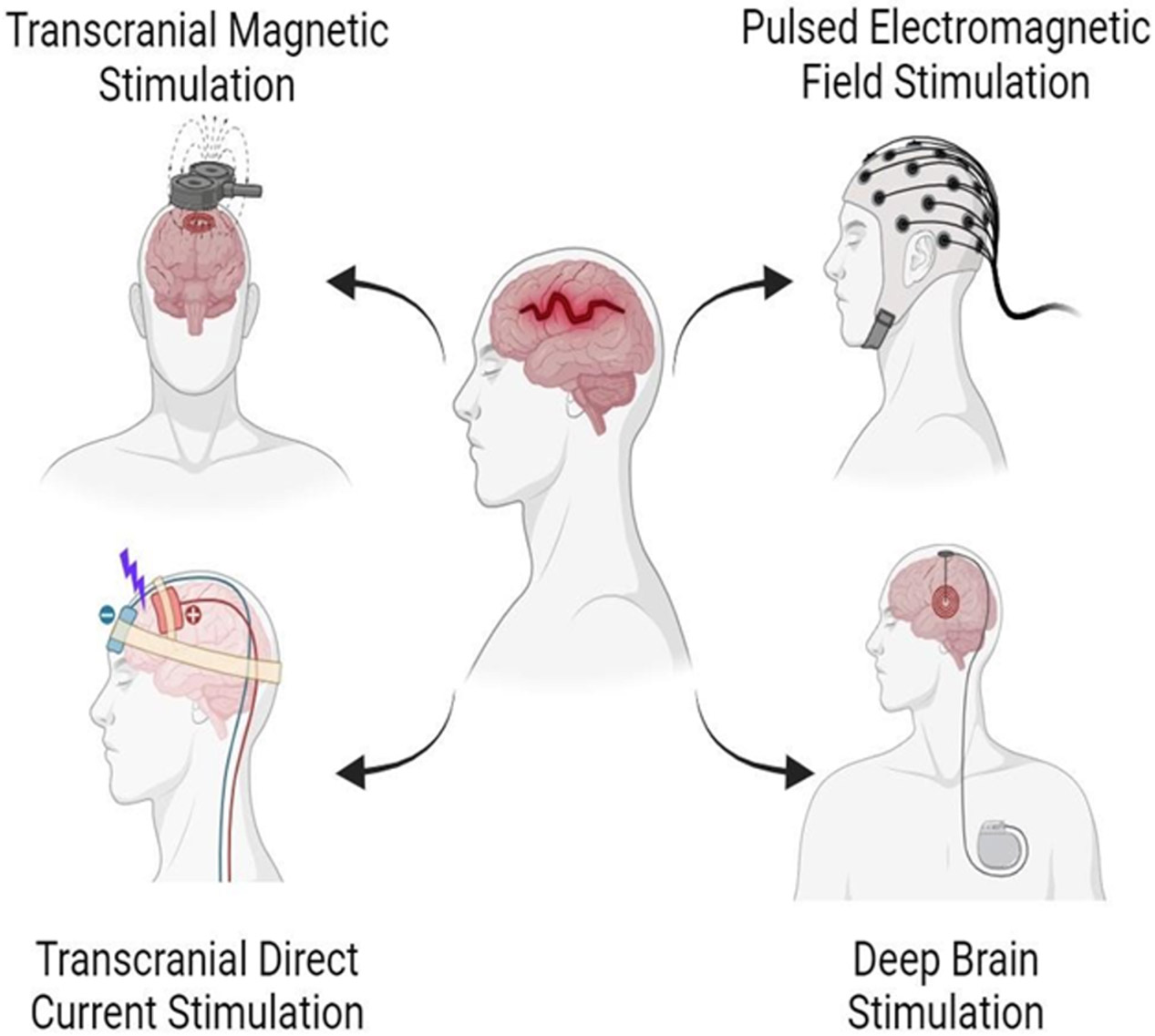
Simplified overview of four stimulation methods described in this article. Non- invasive procedures: Transcranial Magnetic Stimulation employs an alternating magnetic field to stimulate the damaged brain area. Pulsed Electromagnetic Field Stimulation are low-frequency fields with a very specific waveform and amplitude. Transcranial Direct Current Stimulation delivers low intensity electrical currents to the brain via scalp electrodes. Invasive procedure includes Deep Brain Stimulation that delivers electrical impulses to specific areas of the brain through an implanted device.

According to the device employed to apply the electrical or magnetic stimulus into the brain, neurostimulation techniques can be classified as noninvasive or invasive. Non-invasive procedures used for TBI treatment include transcranial magnetic stimulation (TMS), pulsed electromagnetic field stimulation (PEMFS) and transcranial direct current stimulation (tDCS), while deep brain stimulation (DBS) has been employed as an invasive methodology (Figure 1). Noninvasive techniques in particular, modulate neuronal activity without the need for surgical procedures which are costly, require highly trained and experienced personnel, and leave a wound to be managed [50]. Besides, noninvasive techniques can also be safely administered amid situations in which outpatient clinic or rehabilitation center visits are not feasible. Invasive techniques imply the use of surgery to implant an electrode to perform stimulation. In general, the electrical stimulation of the brain deeply influences electrophysiology through modulation of neuronal signaling, not only in the short-term, but also in facilitating or attenuating long-term modifications on a cellular level. The development of synapses is crucial for post-traumatic regeneration and recuperation of high-level cognitive abilities like learning and memory formation, impaired as consequence of TBI [51]. Some of the mechanisms elicited by these stimulatory techniques will be described in the following sections.

## Transcranial Magnetic Stimulation (TMS)

TMS is based in the application of a magnetic field that is generated by a coil and produces a short-lasting electrical current pulse into the brain, mainly in the cerebral cortex, where neurons are stimulated [52]. During the treatment session, the magnetic coil is positioned tangentially to the skull of the subject, leading to the formation of a magnetic field that can penetrate the skull. When stimulation is applied in the form of pulses, the rapid changes in the magnetic field create electrical currents in the brain, which in turn lead to excitation or inhibition of electrical activity, depending on the frequency of stimulation [53]. On the other hand, the selection of different geometric designs and materials define the depth that the magnetic field penetrates the brain as well as the size of the stimulated area. In this sense, circular coils stimulate larger volume of neuronal tissue resulting in greater penetration depth, and designs based on two circular coils positioned next to each other, like a number eight shape, allow for more selective stimulation but is less penetrating [54]. According to this, the main limitation of this method is that the electromagnetic field created by the coil rapidly decreases in strength with increasing distance. But one of the advantages is that, besides local effects, TMS also has remote neurophysiological or behavioral effects in regions that have structural or functional connectivity with the targeted brain region [55].

There are three main types of TMS: a single pulse, paired pulse (ppTMS), and repetitive TMS (rTMS). Single-pulse TMS protocols consist of discharges of single pulses often separated by 4–8 seconds (s) intervals. ppTMS consists of repeated pairing (e.g., 90–200 pairs) of stimuli, while rTMS involves combinations of more than two pulses or bursts of stimulation delivered at a fixed frequency of 0.5–20 Hz, with or without interruption by stimulation-free intervals, for durations from several seconds up to 30–40 min. rTMS can modulate cortical excitability beyond the stimulation period and can be used in both motor and non-motor brain regions [56]. A specific form of rTMS is called theta-burst stimulation (TBS) and consists of 50 Hz bursts of triplet pulses repeated at 5 Hz for a total of 600–1800 pulses. TBS can be intermittent (iTBS), when applied in a pattern of 190 s of stimulation in a 2 s-on/8 s-off scheme; or continuous (cTBS), when administered during a 40 s of continuous stimulation. iTBS and cTBS produce more lasting increased and decreased cortical plasticity, respectively, compared to those obtained with standard rTMS protocols [57]. Regarding the frequency applied, rTMS can be considered as low- frequency, when the stimulation occurs at frequencies lower than 1 Hz, and high-frequency, when it is higher than 5Hz. Low-frequency rTMS reduces neuronal excitability, whereas high-frequency rTMS increases cortical excitability [58].

Neuromodulation of brain circuits damaged by mTBI has emerged as a promising tool for addressing persistent post-concussion symptoms, such as balance and dizziness issues, depression, and headache. TMS can also improve cognitive functions such as recall ability, neural substrates, and performance on neuropsychological tests [59]. However, the efficacy of TMS to improve post-TBI symptoms has shown great variability in clinical scenarios. The small size of patient cohorts and their heterogeneity in terms of TBI phenotype (mild, moderate or severe; recent or old); the variability of protocols applied (anatomical area stimulated; frequency); the endpoint of the studies; primary and secondary variables to be evaluated (mood, motor impairment, pain, cognition, etc.) among other sources of variation, have made difficult to obtain crystal clear results when comparing TMS and control groups. Nonetheless, the results in preclinical studies have been very promising and crucial in describing the molecular mechanisms elicited by TMS after TBI, paving the way to perform new clinical studies. It has been described that TMS simultaneously activates pre-synaptic and post-synaptic neurons, modulating cortical excitability beyond the simulation period [60]. High-intensity repetitive magnetic stimulation (rMS) induced an LTP response in organotypic rodent hippocampal slice cultures *in vitro* with a duration of 2–6 h. This LTP took place at the excitatory synapses on proximal dendrites, in association with the remodeling of small dendritic spines populations on CA1 pyramidal neurons and increased receptor clusters. rMS-induced action potentials in the presynaptic neuron resulted in the release of glutamate into the synapse, whereas simultaneous depolarization of the post-synaptic dendrite by rMS activated voltage-gated calcium channels and removed the magnesium block from the NMDA receptor. As a result, AMPA receptors accumulate on the post-synaptic cell, resulting in a reinforcement of the synapse and LTP-like effects. Whereas a rapid post-synaptic increase in calcium ion content induces LTP, the small and slow flow of calcium ions induces LTD. Low frequency rTMS (≤1 Hz), as well as cTBS, typically induce inhibition or LTD response by altering the efficacy of the excitatory synapse, through altered AMPA receptor trafficking [61].

According to the pattern applied, rTMS modulates the inhibitory expression of the immediate early genes c-fos and zif268, enzymes GAD65 and GAD67 involved in gamma-amino- butyric acid (GABA) synthesis, and calcium-binding proteins [62]. Stimulation patterns that increase cortical excitability, such as 10 Hz and iTBS stimulation, induce Ca^2+^-dependent signaling, depress inhibitory circuits by reducing parvalbumin expression in fast-spiking interneurons and destabilizing GABA receptors to reduce GABAergic synaptic strength. In contrast, inhibitory protocols like 1 Hz and cTBS alter calbindin expression [62]. Ca^2+^ activates calmodulin dependent kinase II and calcineurin, that regulate the initiation and maintenance of LTP and LTD, respectively. Ca^2+^ also regulates NO through calmodulin binding and neuronal NO synthase activation, which regulates synaptic plasticity and network function. Calcium signaling due to rTMS-induced neuronal activity increases calcium-dependent kinase cascades, leading to immediate-early gene expression that promote long-term functional and structural modifications to neurons [63].

**Figure 2:**
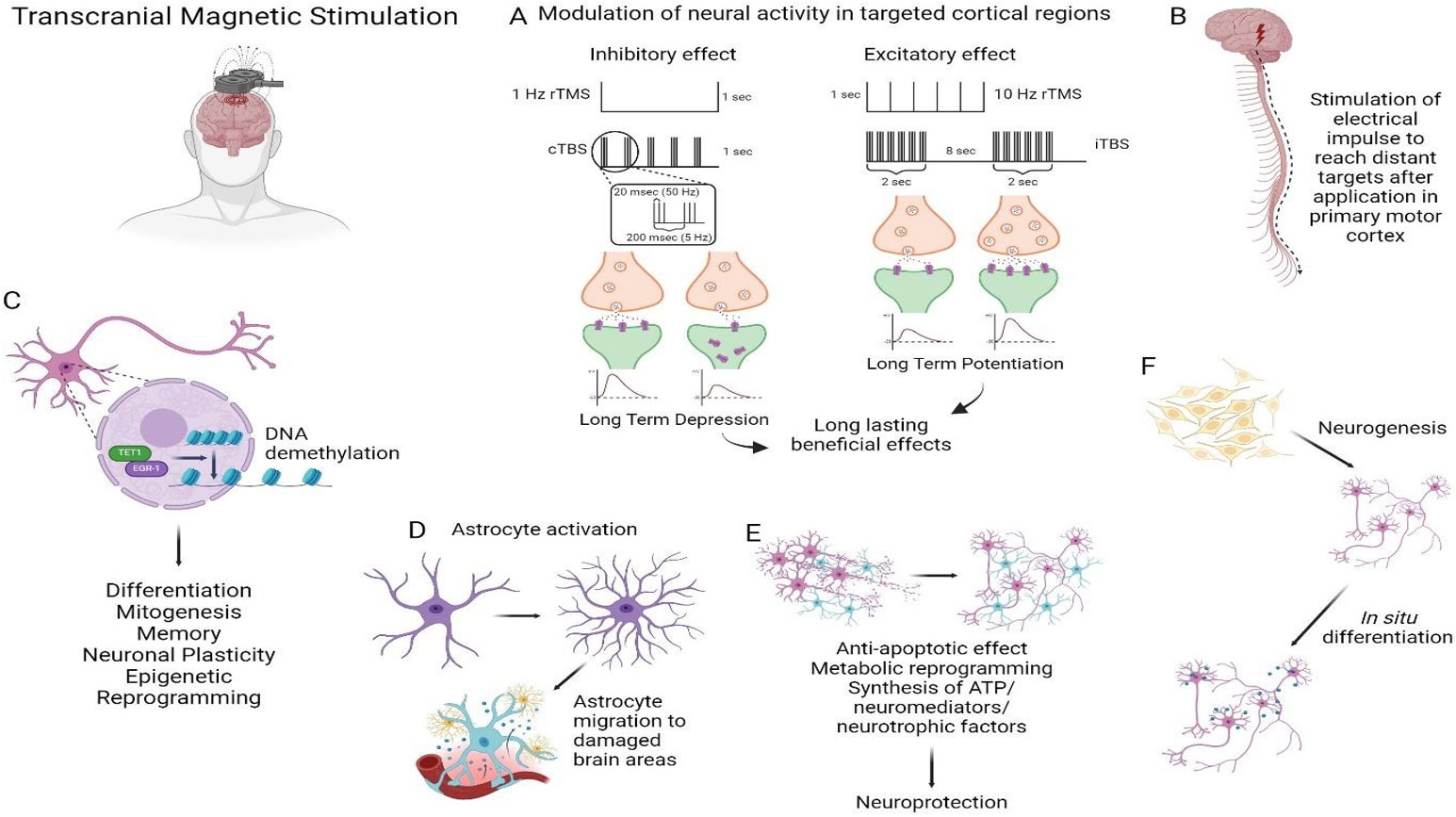
Transcranial Magnetic Stimulation (TMS) mechanisms of action. (A) Modulation of neural activity can be achieved by stimulating long term depression (LTD) through the application of low-frequency repetitive TMS (1 Hz) or continuous thetaburst stimulation (cTBS) or by promoting a long-term potentiation (LTP) through high-frequency repetitive TMS (> 5 Hz) or intermittent thetaburst stimulation (iTBS). (B) Electrical impulse applied to the primary motor cortex travels down the corticospinal tract to stimulate targeted organs and muscles. (C) TMS promotes differentiation, mitogenesis and neuronal plasticity through DNA demethylation of promoter regions of targeted genes. (D) Stimulation of astrocyte activation and migration to damaged brain areas. (E) TMS increases ATP levels, promotes synthesis of brain-derived neurotrophic factor (BDNF) and reduces the expression of pro-apoptotic genes. (F) TMS promotes neurogenesis in targeted areas and *in situ* differentiation of resident neurons. Created with BioRender.com

Besides calcium, TMS can also modulate the activity of other ion channels, such as potassium and chlorine channels, which can further promote TMS-induced changes in neural activity [64]. The molecular effects of TMS are depicted in Figure 2. TMS can induce the activation or inhibition of voltage-gated ion channels and other membrane-binding proteins, including neurotransmitter receptors [65]. Besides, this treatment enhances the secretion of brain-derived neurotrophic factor (BDNF), a potent trophic factor implicated in synaptic plasticity, regeneration, the migration and differentiation of neurons, the growth of dendrites and axons, and neuroprotection. BDNF is also necessary for learning and memory due to its supporting role in neuronal growth and in driving AMPA receptors to synapses [66]. Different studies have shown that TMS increases BDNF levels in the hippocampus, and in the prefrontal cortex and other brain regions in humans, although polymorphisms of this gene affect the way how patients respond to therapy [67]. High frequency TMS is linked to increased ATP level and enhanced expression of microtubule-associated protein 2 (MAP-2) [68]; increased glucose metabolism and significant reduction of the number of caspase-3 positive cells [69]. Also, low frequency stimulation has been associated to an enhanced Bcl-2 and reduced Fas expression, which denotes an activation of anti-apoptotic mechanisms [70]. TMS increased neurogenesis in the hippocampal dentate gyrus subgranular zone, important for neural circuit plasticity [71] and promoted the *in situ* differentiation of neurons in the subventricular zone into dopamine-producing neurons, which associated with a motor recovery [72]. High frequency stimulation had a positive effect on the differentiation and growth of neural stem cells in the neonatal rat *in vitro* and increases newborn neurons, promoting their differentiation and survival [73]. At the systems levels, TMS alters the functioning and interconnections of extensive neural networks by changing the excitability of neurons and the synaptic connectivity between them. TMS selectively modifies the excitability of specific brain regions, resulting in changes in the functioning of interconnected regions and ultimately affecting the activity of the entire network [74]. For example, an increased cortical plasticity following rTMS correlated with the modulation of inhibitory cortical circuits and/or BDNF up-regulation in one or more brain regions [75]. rTMS also activates astrocytes and induces their ability to migrate toward the CNS damaged area [76].

## Pulsed electromagnetic field stimulation (PEMFS)

**Figure 3:**
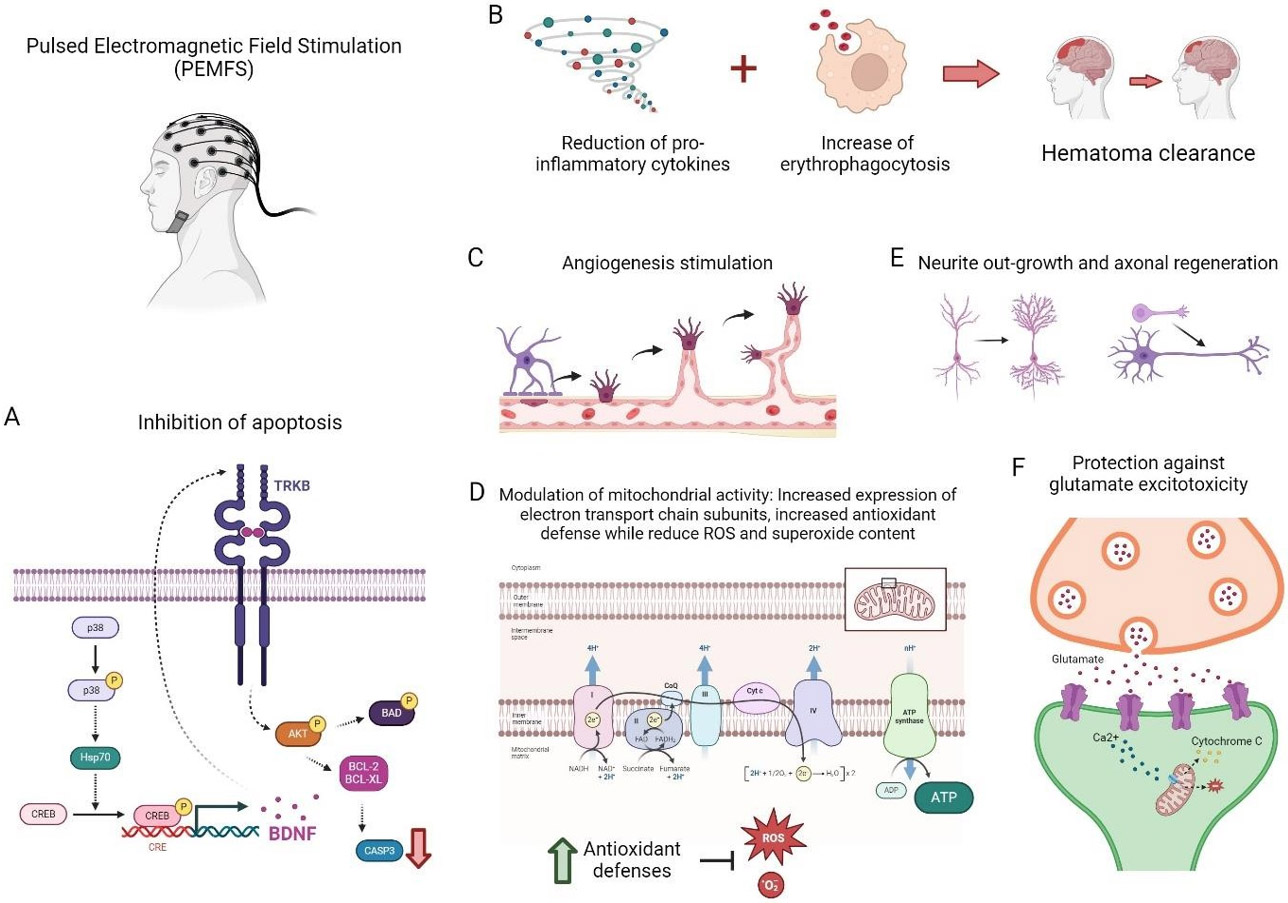
Mechanisms of action of Pulsed Electromagnetic Field Stimulation (PEMFS). (A) Inhibition of apoptosis and stimulation of brain-derived neurotrophic factor (BDNF) via CREB/p38 activation. (B) Reduction of hematoma volume by decreasing the expression of pro-inflammatory cytokines and increase erythrophagocytosis by microglial cells. (C) The pro-angiogenic effect elicited by PEMFS is characterized by an increased vascular growth rate and increased capillary density. (D) Prevention of Reactive Oxygen Species (ROS) production by increasing superoxide dismutase activity. (E) Promote of neurite out-growth and axonal regeneration. (F) Reduction of glutamate-induced excitotoxicity by increasing cell viability and decreasing lactate dehydrogenase release. Created with BioRender.com

Electromagnetic field (EMF) is a magnetic field produced by moving electrically charged particles and can be viewed as a combination of electrical and magnetic fields. EMF can be divided into stationary magnetic fields and time-varying magnetic fields. Stationary has the same direction and magnitude in time because the electrical component is suppressed or has a 0 Hz frequency. The most common sources of EMF are permanent magnets or electromagnetic coils with direct current. In time-varying magnetic fields, the magnetic field intensity and treatment strategy (time and days of exposure), as well as the frequency and waveform can be altered and, as a consequence, the magnetic field varies over time. This modality can be classified in pulsed electromagnetic field (PEMF) and sinusoidal electromagnetic field (SEMF). PEMFs could be asymmetric, biphasic, quasi-rectangular, or quasi-triangular in shape [77]. SEMFs, on the other hand, follow a sinusoidal waveform. One of the advantages of EMF treatment resides in that this is a non-invasive method and can be applied without the need for anesthesia, nor wounding the patient [77]. PEMFS are usually low-frequency fields with a very specific waveform and amplitude and are characterized by a constant variation in the amplitude of the magnetic field over time. The pulsed magnetic field is generated by a wire coil wherein electric current circulates. Such current is responsible for the generation of the pulsed magnetic field, which in turn, induces a time-varying secondary electrical field within the exposed tissue [78]. PEMFS differs from TMS in that PEMFS devices emit low-frequency electromagnetic waves, like those found naturally in the environment, while in TMS higher frequency magnetic pulses are applied. According to the elicited mechanism of action, it has been suggested that PEMFs might be used as a therapeutical approach for treating musculoskeletal disorders [79] and for promoting proliferation and migration of fibroblasts and keratinocytes during wound healing and regeneration [80]. Besides, it has been reported as neuroprotective tools due to their antiapoptotic effect against ischemic cell death. Specifically, in a distal middle cerebral artery occlusion in mice, PEMF significantly influenced expression profile of pro- and anti-inflammatory factors in the hemisphere ipsilateral to ischemic damage. In the same experimental model of cerebral ischemia, a significant reduction of infarct size mediated by a chronic treatment of PEMFs has been oberved compared to controls, that is why it has emerged as a potential alternative to the pharmacological protocols in ischemic diseases [78]. The mechanisms of action elicited by EMF application include interaction with the cellular membrane, effects in Ca^2+^ intracellular concentration, and the production of free radicals; enhancement of NO production, increase and/or decrease of superoxide production, and inhibition of apoptosis. In addition, EMF reduces edema and inflammation [78], cornerstones in the development of neurological complications after TBI. Molecular mechanisms promoted by PEMFS are summarized in Figure 3.

In particular, PEMFs have been shown to exert protective effects in different animal models of neurological disorders, reducing infarct size and neuroinflammation, decreasing pro-apoptotic mediators and increasing pro-survival molecules and promoting angiogenesis and modulating microcirculation [78]. The pro-angiogenic effect is characterized by an increased vascular growth rate and increased capillary density. The potential mechanisms consist of promoting vascular endothelial cell proliferation, migration, and tube formation, and increasing the expression level of vascular endothelial growth factor, fibroblast growth factor 2, angiopoietin 2, and other angiogenic growth factors. Additionally, PEMF has an impact on the activation of voltage-gated calcium channels [81]. In a mouse model of intracerebral hemorrhage (ICH) and cultured BV2 cells, PEMF decreased the hematoma volume and the expression levels of proinflammatory factors after ICH. Moreover, PEMF enhanced the erythrophagocytosis of microglia via CD36. The downregulation CD36 with genistein blocked the effects of PEMF-induced hematoma clearance and anti-inflammatory effects. Thus, the PEMF-mediated promotion of neurological functions may, at least partly, involve anti-inflammatory processes and hematoma clearance [82].

PEMFs treatment significantly increased CREB via p38 activation and restored the hypoxia-reduced BDNF levels [83]. Several results from *in vitro* experiments suggested that PEMF reduced glutamate-induced excitotoxicity by increasing cell viability and decreasing lactate dehydrogenase release [84]. PEMF increases the function of endogenous adenosine by the upregulation of A2A and A3 receptors in neuronal cells, resulting in neuroprotection [78]. Other neuroprotective effects include cell proliferation and differentiation, enhanced neurite outgrowth and increased microvascular perfusion and tissue oxygenation [85]. PEMF exposure counteracted hypoxia damage, significantly reducing cell death and apoptosis in the human neuroblastoma-derived SH-SY5Y cells and the rat pheochromocytoma PC12 cells. In these cell lines, PEMFs inhibited the activation of the hypoxia-inducible factor 1a, the master transcriptional regulator of cellular response to hypoxia, decreasing hypoxia-induced ROS generation These data are similar to those obtained in human neuroblastoma cell line where PEMFs prevented H2O2-induced ROS production by increasing superoxide dismutase activity [86] . Besides its effect on neuronal cells, PEMF has also shown to reduce hypoxia-induced ROS and the release of TNF-α, IL-1β and IL-6 in LPS-activated N9 microglial cells [87].

Recently our group demonstrated the effectiveness of EMF stimulation with a fixed frequency application in an animal model of TBI based on controlled cortical impact in swine [88-90]. Animals were stimulated using a proprietary non-contact, non-invasive induction sensors with a dual layer Mu- metal and interlaced copper mesh helmet, to apply a sinusoidal wave individualized at 2.5 Hz with a 500 mV positive offset at 1 V during the first 10 days after the injury. After that, the settings were modified to another individualized frequency of 5.5 Hz, 500 mV positive offset and 1 V for stimulation to complete three weeks of treatment [88-90]. According to our results, there were more viable glial and neural cells and fewer apoptotic cells within the cortical areas of the stimulated subjects compared to the non-stimulated swine, which had more apoptotic cells and higher degrees of cellular injury [90]. The findings in the treatment group supported a significant reduction in the expression of the NLRP3 inflammasome molecules and pro-inflammatory cytokines in the injured cortical area, and of circulating levels of TBI markers such as ubiquitin carboxyterminal hydrolase [91]. RNA sequencing results suggested that the differentially expressed genes after the treatment were associated with immune cell infiltration, myelination, reactive oxygen species regulation, thyroid hormone transportation, cell proliferation, and cell migration, all of them contributing to beneficial effects of EMF stimulation during the repair process following TBI [92, 93]. The neuroprotective effects of PEMF have been demonstrated on patients with depression [94] and with Parkinson's disease [95]. Unfortunately, there are limited reports on the investigation of PEMF effect in TBI. Miller et al. carried out a study with a small (n=7) group of TBI patients that received 5 weeks of daily 30 minutes T-PEMF treatment with evaluation after 2 and 5 weeks and 3 months after ending treatment. The study concluded that 5/7 patients had a reduction in symptoms overall up to 61% (2%-61%), based on the Rivermead Post-Concussion Symptoms Questionnaire. Although with limitations, the authors considered that the treatment showed promise and feasibility criteria for compliance and tolerability were met [96].

## Transcranial Direct Current Stimulation (tDCS)

Another type of noninvasive neurostimulation is tDCS, where electrodes are placed directly on the subject’s scalp to generate an electric current by delivering low amplitude (<2 mA) over a short period (<30 min) between anode and cathode, that is intended to penetrate the skull and modulate neural activity. This method has been shown to modulate cortical excitability, producing changes of up to 40% that can last for between 30 and 120 minutes [97]. There are several parameters that defines the polarity-effects and cortical excitability changes of tDCS, for example, configuration of electrodes; targeting the cortical area to be stimulated; polarity of stimulation; intensity of stimulus; duration and number of sessions. Regarding the polarity, it has been shown that anodal stimulation depolarizes the neuronal membrane and increases motor-cortex excitability and motor-evoked potential (MEP) amplitude by about 40%, whereas cathodal hyperpolarizes the neuronal membrane and decreases motor-cortex excitability and MEP amplitude. This change of the resting membrane threshold induces a sustainable response in the form of LTP or LTD [98]. Besides the local changes in membrane polarity, the direct electrical current can reach and modulate subcortical and deeper structures that are connected to the stimulated cortical area such as the red nucleus, medial longitudinal fascicle and thalamus [99]. In general, during cathodal tDCS, the cathode is placed over the area of interest and in anodal tDCS, the anode is placed over the target area. The mechanisms underlying the “after-effects” of tDCS are attributed not exclusively to the changes of electrical neuronal membrane potential, but also to the changes on NMDA and GABA receptors. tDCS interferes with brain excitability through modulation of intracortical and corticospinal neurons resulting in modulatory effects of NMDA and GABAergic receptor efficacy. The anodal tDCS may enhance the excitatory synaptic transmissions by changing the balance between glutamate and GABA activities, with enhancing the effect on glutamatergic neurons by reducing GABA activity. tDCS enhances or reduces calcium influx via NMDA receptors and voltage-gated calcium channels and, in turn, the amount of intracellular calcium controls the induction of both LTP and LTD [100]. Neuromodulatory effects also include changes in BDNF expression and its release into the synaptic cleft. Podda et al. demonstrated that tDCS increased hippocampal LTP, learning and memory and these effects were associated with enhanced acetylation of BDNF promoter, expression of BDNF exons I and IX and BDNF protein levels. The stimulation of this neurotrophic factor was mediated by CREB phosphorylation, pCREB binding to BDNF promoter I and recruitment of CBP to this regulatory sequence [101]. Likewise, in anodal tDCS, tropomyosin-receptor kinase (Trk) receptors are attracted to the synapse where they induce later phase LTP and facilitate the opening of NMDA receptors. It has been demonstrated that inhibition of acetylation and blockade of TrkB receptors hinders anodal tDCS effects at molecular, electrophysiological and behavioral levels. On the contrary, in cathodal tDCS they promote the later phase LTD [101]. tDCS may also involve the regulation of various neurotransmitters, such as GABA, dopamine, acetylcholine, serotonin, epinephrine and norepinephrine [102].

The increased excitability of local neurons by anodal stimulation increases blood flow around the stimulation site, inducing subsequent metabolic changes. Merzagora et al. observed higher levels of oxygenated hemoglobin under the anodal electrode and compared to those for the cathode, and this is thought to reflect the ability of anodal stimulation to induce metabolic changes among neurons [103]. Another group observed a restoration of impaired cerebrovascular reactivity to hypercapnia, improved cerebral blood flow and tissue oxygenation, which is a key factor in brain metabolism associated with brain damage in the acute phase of TBI [104]. The tDCS may reduce pain by modulating neuroinflammation, possibly through the stimulation of brain immune cells, such as mast cells and glial cells, to regulate pro-inflammatory cytokines release. tDCS reduces the activation of microglia, decreases the synthesis of pro- inflammatory cytokines TNF-α, IL-6 and IL-1β and increases the levels of anti-inflammatory mediators like IL-1α, IL-10 and IL-4 [105]. Increases in growth associated protein-43, a protein synthesized during axonal growth, and MAP-2, involved in dendritic remodeling along with an increase in dendritic density, has been reported in stimulated brain structures in rats with ischemic lesions, which further suggests that anodal tDCS may have neuroprotective as well as neurorestorative properties. *In vitro*, direct current electric field accelerates and polarizes the migration of lymphocytes, monocytes, neutrophils, macrophages, and polymorphonuclear cells [106].

**Figure 4:**
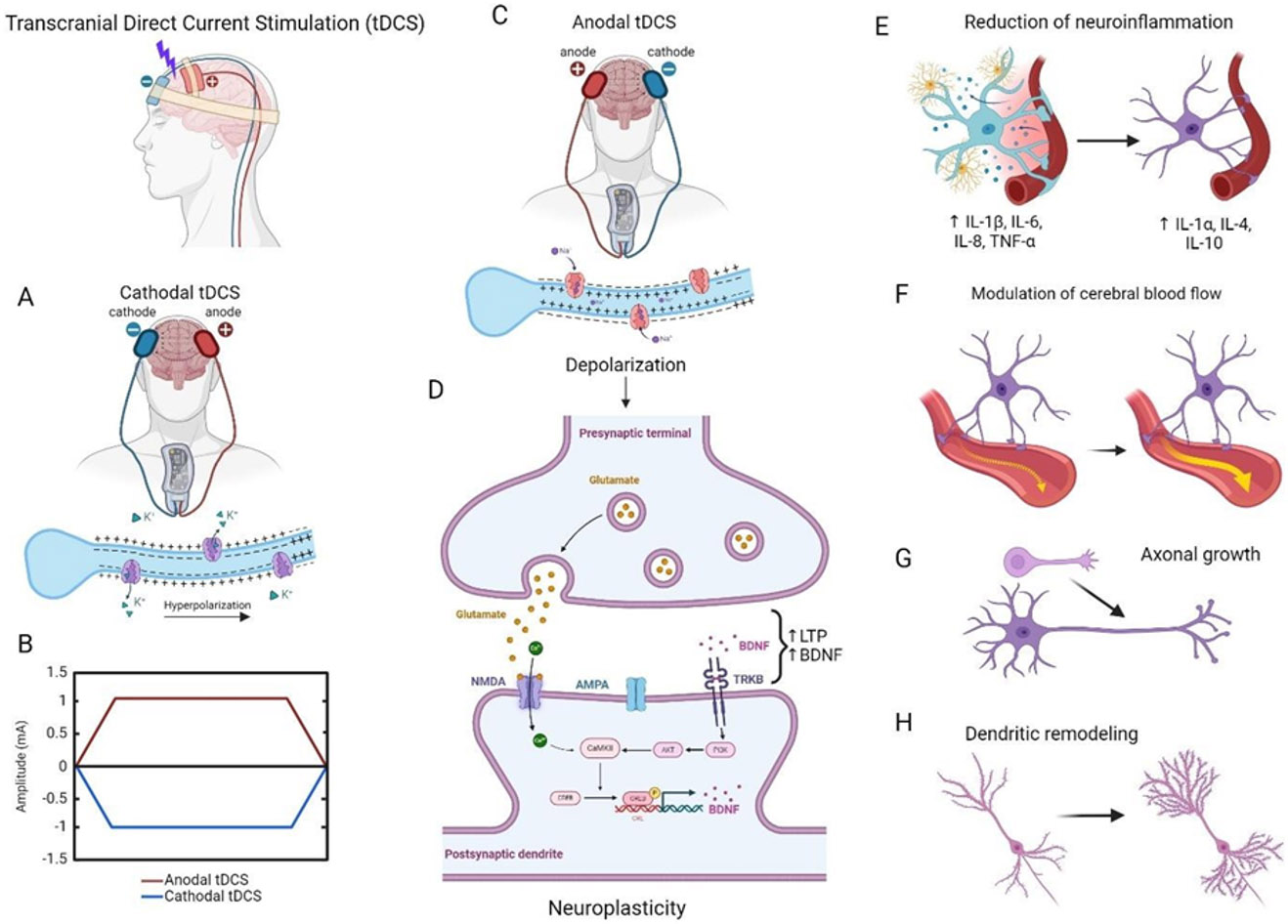
Mechanisms of action of Transcranial Direct Current Stimulation (tDCS). (A) Cathodal tDCS hyperpolarizes the neuronal membrane and decreases motor-cortex excitability and motor-evoked potential amplitude, (B) Anodal (red) and cathodal (blue) tDCS waveforms, (C) Anodal stimulation depolarizes the neuronal membrane and increases motor- cortex excitability and motor-evoked potential, (D) Anodal tDCS enhances the excitatory synaptic transmissions by increasing calcium influx via NMDA receptors. Neuromodulatory effects also include changes in brain-derived neurotrophic factor (BDNF) activity, (E) tDCS reduces the activation of microglia, reduces the expression of pro-inflammatory cytokines and promotes the expression of anti-inflammatory cytokines, (F) tDCS increases regional cerebral blood flow, (G) tDCS promotes axonal growth, and (H) tDCS induces dendritic remodeling. Created with BioRender.com

A summary of molecular mechanisms caused by tDCS can be observed in Figure 4. In animal models of TBI, tDCS application was associated to an improvement in impulsivity, behavioral and spatial memory impairment, balance and posture [107]. In the clinical scenario, it is necessary to consider the heterogeneity of brain injury sites within TBI population. That is why the results obtained from tDCS application may vary substantially and would be reflected in brain activity changes that might be assessed via neuroimaging and neuropsychological tests. The intra- and inter-individual variabilities in “ideal” parameters for electrical current application, stimulation targets, and responses are some of the major concerns, slowing down the widespread use of tDCS in clinical settings. Among the factors that influence the response to tDCS are conductivity of the underlying tissues, registration procedure errors, anatomic variations, functional connectivity, and inter-individual variability (e.g., age, gender, hormones, neurotransmitter levels, neuroanatomy, and sex hormone differences [108]. Despite all that, TBI patients have shown improved motor deficiencies, diminishing of attention deficits, and an improvement in other clinical parameters like coma recovery and cognitive outcomes [109].

## Deep Brain Stimulation (DBS)

Invasive stimulation methods, such as DBS, may achieve higher precision and efficiency by bringing the stimulation electrodes closer to the desired area. In this case, the stimulation electrodes are implanted stereotactically at specific targets in the brain. The electrodes are connected to a pulse generator, which is a pacemaker-like device that is implanted under the skin in the chest wall and typically located beneath the collarbone. The clinician establishes the stimulation parameters through a computer, which communicates with the implanted pulse generator via a transcutaneous connection. Stimulation parameters include single pulse or continuous stimulation, amplitude, voltage, polarity, frequency, pulse width, pulse shape, rhythm and cause diverse electrical effects [110]. Stimulation electrodes are often implanted bilaterally and commonly have multiple metal contacts, which can be used both as anodes and as cathodes. In bipolar configurations, an electrical field is generated between two adjacent contacts, allowing for a concentrated electric field and thus a higher precision. The electrical excitability of neural tissue around the electrode and the effects of stimulation are determined by several factors: (i) the proportion of gray and white matter structures (i.e., cell bodies or axons); (ii) the type of ion channels on the cell membrane of a soma or axon; (iii) the diameter and degree of myelination of axons; (iv) the orientation of axons in relation to the electrode; (v) the distance of the stimulated structure to the electrode; and (vi) the microenvironment (astrocytes and microglia) [111]. The electrode implanted into the brain and polarized to a negative potential (cathode) redistributes charged particles (such as Na^+^ and Cl^−^ ions) throughout the extracellular space. This redistribution creates an electric field that can manipulate the voltage sensor of sodium channel proteins present in the membrane of neurons. The opening of sodium channels can generate an action potential, which typically initiates in the axon. Stimulation- induced action potentials then propagate in both the orthodromic (toward the synapse) and antidromic (toward the soma) directions to the axon terminals of the neuron. This leads to complex patterns of activity with the net result that DBS may have both excitatory and inhibitory effects on neuronal activity [112]. The overall outcome will be a silenced neuronal firing of the target structure, with an increased synaptic output of the stimulated axons near the electrodes [111].

DBS also stimulates astrocytes. These glial cells release neurotransmitters and neuromodulators that influence neurons, which might contribute to the therapeutic long-term effect of DBS. Electrical stimulation can activate astrocytes via calcium ion influx, and in turn activated astrocytes release glutamate and adenosine and increase the probability of neurotransmitter release from excitatory or inhibitory presynaptic terminals [113]. From an astrocytic point of view, the local inhibitory effect of DBS can be explained by a direct stimulation of astrocytes to release ATP, which subsequently leads to an inhibition of synaptic transmission through the action of adenosine on post- and pre-synaptic A1 receptors. Glia in general and astrocytes in particular may be involved in the effects of DBS in three different ways: astrocytes can be triggered by electrical stimulation, change the cerebral blood flow and release ATP and glutamate, both important neuromodulators and regulators of neuronal synaptic networks. Based on this, DBS-induced modulation of network activity is partially due to astrocytic gliotransmission [114].

In the pre-clinical scenario, DBS was initially applied in 2013. With the possibility to target small and specific areas as well as deeper regions of the brain, DBS can be used to treat a wide variety of sequelae related to TBI such as the loss of cognitive and motor function, bladder dysfunction and disorders of consciousness. In a wide number of studies, animals received stimulation in multiple sessions over several days or weeks, starting shortly after injury or several days after the induction of TBI. Therapy of cognitive deficits was shown to be effective in the acute, subacute and chronic phases of TBI. It was shown that DBS improved motor function, voiding efficiency and spatial working memory, as well as attenuated hippocampal theta activity after TBI. Researchers also observed a mediation of anti-apoptotic and anti-inflammatory effects after DBS, while others confirmed that it may promote wakefulness.

Although very promising results have been obtained, most of the studies did not investigate the persisting effects of DBS. Only in one study the beneficial effects were observed regarding their task-matched stimulation approach on spatial memory that persisted 10 days after stimulation cessation. A detailed summary of the preclinical results obtained after the application of DBS and other neurostimulation techniques in TBI models can be accessed [115]. DBS has been approved for the symptomatic treatment of Parkinson’s disease, essential tremor, obsessive compulsive disorder and some cases of severe epilepsy in humans [116]. It has been hypothesized that DBS has potential use in the treatment of higher-order cognitive dysfunction and disorders of consciousness in patients with TBI [117], although there are scarce examples in the clinical arena. Central thalamic DBS induced the recovery of a patient in a persistent minimally conscious state, although it has been demonstrated that the effectiveness of the treatment depends on the severity of the trauma [118]. In a recent phase 1 randomized feasibility study regarding thalamic DBS in TBI patients, six participants with moderate to severe TBI, who were between 3 and 18 years post-injury, underwent surgery with electrode placement guided by imaging and subject-specific biophysical modeling to predict activation of the central lateral and the associated medial dorsal tegmental tract. DBS was administered for 12 hours a day for three months. The primary efficacy measure was improvement in executive control indexed by processing speed on part B of the trail-making test. Five participants completed the study. Processing speed on part B of the trail-making test improved 15% to 52% from baseline, exceeding the 10% benchmark for improvement in all five cases. The researchers concluded that DBS can be safely applied in the selected anatomical area and may improve executive control in patients who are in the chronic phase of recovery [119].

## Conclusions

TBI continues to be one of the main causes of disability and mortality, not only in adults, but also in children and young people. The large number of sequelae associated to trauma encourages the research and development of drugs and technologies capable of reducing the complications derived from the injury, to increase the quality of life and extend the life expectancy of TBI patients. In this sense, the use of neurostimulation procedures, based on the application of electric or magnetic fields, is emerging as a novel approach, which in some cases even has the advantage of being able to be administered at home. Despite the positive results obtained so far, there are several limitations that need to be resolved in advance for obtaining the approval of regulatory authorities and extend the procedures as regular treatments for patients. In the pre-clinical scenario, it is necessary to continue digging into the underlying mechanisms concerning precise electrical stimulation of specific brain areas. It would be also needed to find a consensus about the most effective stimulation parameters and time frames for a variety of impairments. But most important, it is critical to investigate the long-term effects of the different treatments, as current experiments often lasted for less than one week, and animals were often sacrificed directly after an experiment or shortly after stimulation was terminated. In the clinical arena, the limited sample size of study population results in a lower statistical power, limiting the generalizability of the conclusions and the reliability of the therapeutic effects of stimulation techniques in TBI patients. Hence, future studies may consider increasing the sample size appropriately. Moreover, the stimulation protocols, with respect to total number of pulses, duration frequency and intensity of stimulation, location and number of sessions delivered, timing of the concomitant neurorehabilitation, are highly heterogeneous. Therefore, estimating the real effectiveness and reproducibility of a given procedure is very difficult. As in pre-clinical studies, it is also necessary to conduct follow-up testing at longer intervals, to appreciate possible prolonged effects following treatments. The complex anatomy and functioning of the human brain, the different types of cells that interact with each other, the physical, chemical and biological mechanisms that are triggered by damage and how different treatments can modulate them constitute areas yet to be explored. Understanding the various components of brain stimulation response is still necessary. The results obtained so far in this field have been an encouraging starting point that paved the way for future research.

## Figures and Tables

**Figure 5:**
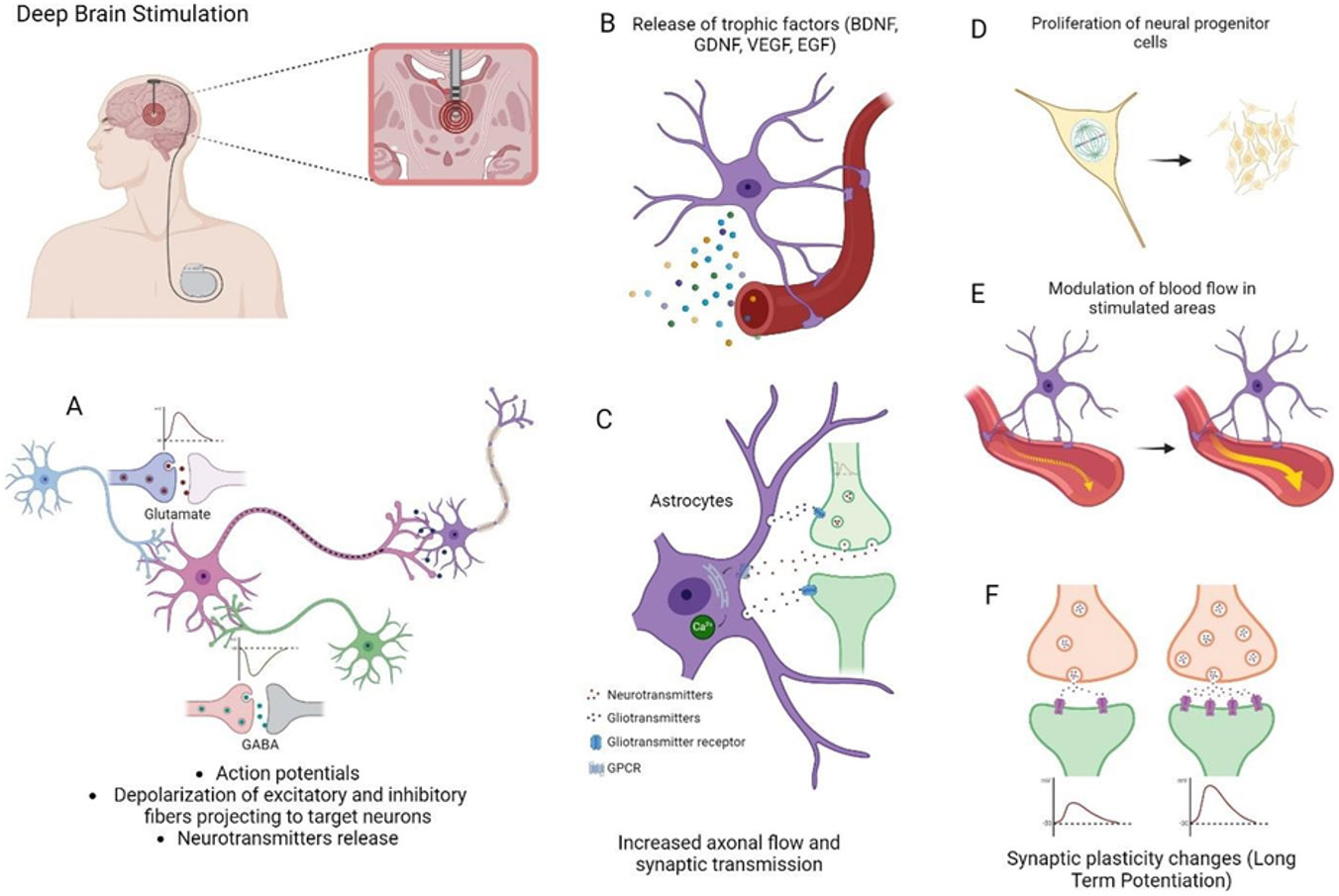
Mechanisms of action of Deep Brain Stimulation (DBS). (A) DBS causes axonal action potentials and depolarization of inhibitory and excitatory fibers projecting to target neurons, (B) In response to DBS activated astrocytes release calcium and trophic factors, (C) Neurotransmitters released by depolarized neurons reach astrocytic Gq protein-coupled receptors (Gq-GPCRs), resulting in an increase in intracellular Ca^2+^, which activates gliotransmitter release, (D) significant increase in neural progenitor cells in the stimulated areas, (E) Modulation of blood flow in stimulated areas due to release of gliotransmitters, and (F) DBS leads to changes in neuronal firing rate associated to neurotransmitter release and synaptic plasticity changes like long-term potentiation. Created with BioRender.com

## Key Points, Outstanding Questions, and Challenges

- Traumatic brain injury comprises a first stage of damage following the mechanical impact to the head characterized by laceration, hemorrhages and local necrosis; and a second and most extended phase of damage that includes blood-brain barrier disturbance, glutamate excitotoxicity, neuroinflammation, oxidative and nitrosative stress, mitochondrial dysfunction, cerebral edema, demyelination, and decreased neurogenesis.
- Neuromodulation elicited by the application of electrical or magnetic stimulus is one of the therapeutical approaches currently under investigation aiming to promote neuronal plasticity and functional recovery in patients of neurodegenerative disorders and external injuries like TBI.
- The results from the studies in experimental TBI model using transcranial magnetic stimulation, pulsed electromagnetic field stimulation, transcranial direct current stimulation and deep brain stimulation support enhancement of molecular mechanisms that induce long term potentiation and depression, neurotransmitters and neurotrophic factors synthesis, reduction of pro-inflammatory and pro-apoptotic markers, neurogenesis, and differentiation.
- What are the most appropriate electrical and magnetic parameters during the application of the aforementioned methodologies to obtain a better clinical response in the short and long term in TBI patients?
- In addition to the molecular responses described so far, triggered after the application of neurostimulation techniques, are there other molecular pathways and specific targets involved?
- The ethical implications of using human brain tissues constitute a challenge to overcome in order to evaluate the results of applying these neurostimulation techniques directly to patients, limiting the studies to *in vitro* experiments and animal models that do not always reflect the complexity of the human brain.
